# Reproductive Outcomes of Transferring Blastocysts Derived From Frozen–Thawed Cleavage Embryos: A Systematic Review and Meta‐Analysis

**DOI:** 10.1002/rmb2.12673

**Published:** 2025-08-07

**Authors:** Huy Phuong Tran, Nam Nhat Nguyen, Ngoc Thanh Truong, Tuyet Thi‐Diem Hoang, Trang Nguyen‐Khanh Huynh, Ha Le‐Bao Tran

**Affiliations:** ^1^ Faculty of Biology and Biotechnology University of Science Ho Chi Minh City Vietnam; ^2^ Laboratory of Tissue Engineering and Biomedical Materials University of Science Ho Chi Minh City Vietnam; ^3^ Vietnam National University Ho Chi Minh City Vietnam; ^4^ Hung Vuong Hospital Ho Chi Minh City Vietnam; ^5^ Endocrinology Clinic Hanh Phuc International Hospital Thuận An Binh Duong Vietnam; ^6^ Department of Obstetrics and Gynecology Pham Ngoc Thach University of Medicine Ho Chi Minh City Vietnam

**Keywords:** blastocyst, cleavage embryo, frozen–thawed embryo transfer, IVF

## Abstract

**Background:**

In in vitro fertilization (IVF), transferring frozen–thawed blastocysts is a widely adopted practice. This meta‐analysis aims to evaluate the reproductive outcomes of transferring blastocysts derived from frozen–thawed cleavage embryos (FT‐CDB group) compared to direct frozen–thawed blastocyst (DFB group) transfers.

**Methods:**

We searched the following electronic databases for relevant studies: PubMed/MEDLINE, EMBASE, Scopus, and Web of Science. Studies were included if they compared the clinical and neonatal outcomes of IVF patients receiving either FT‐CDB or DFB transfer with vitrification method. The protocol for this review has been registered in PROSPERO.

**Results:**

A total of seven studies (2057 patients) were included in the analysis. Participants in the FT‐CDB group demonstrated significantly higher odds of achieving clinical pregnancy (OR 1.24, 95% CI 1.03–1.49, *p* = 0.022, *I* ^2^ = 27%), and live birth (OR 1.31, 95% CI 1.08–1.60, *p* = 0.007, *I* ^2^ = 0%) compared to the DFB group. No significant differences were observed in the birth weights of infants between the groups (MD −87.05 g, 95% CI −293.77 to 119.67, *p* = 0.41, *I* ^2^ = 83%).

**Conclusion:**

Transferring blastocysts derived from frozen–thawed cleavage embryos is associated with higher odds of clinical pregnancy and live birth compared to frozen–thawed blastocyst transfers.

**Trial Registration:**

PROSPERO number: CRD42024591620

## 1 Introduction

Advances in assisted reproductive technology (ART) have enhanced pregnancy outcomes for individuals undergoing in vitro fertilization (IVF). Cryopreservation techniques, particularly for cleavage‐stage embryos and blastocysts, have become integral to modern ART practices [1]. By enabling the storage of surplus embryos, cryopreservation increases cumulative pregnancy rates and allows multiple attempts at achieving pregnancy without requiring repeated ovarian stimulation and egg retrieval [2]. Furthermore, it provides flexibility in treatment timing, facilitating the scheduling of frozen embryo transfers (FET) during periods of optimal uterine receptivity [3]. This is especially beneficial in cases where fresh transfers are not advisable, such as in patients at risk of ovarian hyperstimulation syndrome (OHSS) or those with suboptimal endometrial conditions. Additionally, cryopreservation is invaluable for individuals undergoing medical treatments like chemotherapy, as it offers the opportunity to preserve fertility for future family planning [4]. It also supports preimplantation genetic testing (PGT), enabling the selection of genetically healthy embryos, thereby further improving reproductive outcomes.

Cryopreservation of cleavage embryos has long been the most common practice. However, recent advances in laboratory techniques and culture media have introduced extended embryo culture. The shift toward blastocyst‐stage transfers is driven by the higher implantation potential of blastocysts and their improved synchronization with endometrial receptivity [5]. Additionally, culturing embryos to the blastocyst stage allows for better selection of viable embryos, which may enhance reproductive outcomes [6]. Despite these advantages, challenges remain in determining the optimal strategy for embryo cryopreservation and transfer. An emerging clinical question is whether cryopreserving cleavage‐stage embryos, followed by thawing and culturing to blastocysts (FT‐CDB), achieves better reproductive outcomes compared to the transfer of directly thawed blastocysts (DFB). Despite the widespread use of both strategies, there remains a lack of consensus on which approach offers superior reproductive outcomes. Understanding these differences is crucial for optimizing IVF protocols and improving patient outcomes.

This systematic review and meta‐analysis aims to compare outcomes between FT‐CDB and DFB transfers. By evaluating existing evidence, this study seeks to provide a clearer understanding of the relative efficacy of these approaches and inform clinical decision‐making in ART.

## 2 Materials and Methods

### 2.1 Search Strategy and Selection Criteria

We performed a comprehensive search across multiple databases, including PubMed/MEDLINE, EMBASE, Scopus, and Web of Science to identify relevant articles published up to September 30, 2024. The search had no restrictions on language or publication year. Key terms such as “blastocyst,” “cleavage‐stage embryo,” “pregnancy outcomes,” and related phrases were used. Details of the search strategy are provided in Table S1. Study management was facilitated using Endnote (version 21; Clarivate, Philadelphia, PA, USA).

We included studies that met specific criteria: full‐text articles, conference abstracts, and preprints, with a preference for official publications. Two independent reviewers conducted the search, screened studies against the inclusion criteria, and extracted key data. Disagreements were resolved through discussions involving a third reviewer. The study followed PRISMA guidelines. Included studies evaluated pregnancy and embryo outcomes, such as cryosurvival rate, implantation rate (IR), positive hCG rate (PHR), clinical pregnancy rate (CPR), ongoing pregnancy rate (OPR), pregnancy failure rate (PFR), and live birth rate (LBR), comparing the FT‐CDB group to the DFB group. Specifically, cryosurvival rate and IR were calculated per embryo. Other outcomes—including PHR, CPR, OPR, PFR, and LBR—were calculated per patient, reflecting standard clinical reporting. The exposure group consisted of women undergoing FT‐CDB transfers, while the control group comprised those undergoing DFB transfers. Only studies that employed the vitrification method for cryopreservation were included to ensure consistency in the cryopreservation protocol.

Exclusion criteria were as follows: studies comparing fresh embryo transfer cycles and studies not addressing outcomes of interest. The protocol for this systematic review and meta‐analysis was registered with PROSPERO.

### 2.2 Data Extraction

Details including the number of events, total observations, and demographic variables such as country, publication year, maternal age, body‐mass index (BMI), duration of infertility, and endometrial thickness were recorded and organized using Excel.

### 2.3 Data Analysis

The quality of the included articles was evaluated using the Newcastle–Ottawa Scale [7]. For pregnancy and embryo outcomes, effect sizes were calculated by taking the natural logarithm of the odds ratios (ORs). The log OR and its standard error were then used as inputs for the meta‐analysis, with the results displayed in a forest plot showing the ORs for individual studies and the overall summary. For maternal age, BMI, duration of infertility, and endometrial thickness, a comparative meta‐analysis was conducted to determine the mean differences (MDs) between the FT‐CDB and DFB groups for each parameter. Heterogeneity was assessed using Cochran's *Q* test and the *I* ^2^ statistic. A random‐effects model was applied when *p* < 0.1 in Cochran's *Q* test or *I* ^2^ > 50%; while a common‐effects model was used otherwise. Egger's asymmetry test was performed to assess publication bias, with *p* < 0.05 indicating potential bias. Statistical significance was defined as *p* < 0.05, and all analyses were conducted using R software (version 4.0.2; R Foundation for Statistical Computing, Vienna, Austria).

## 3 Results

### 3.1 Study Selection

Figure S1 illustrates the flowchart outlining the study inclusion process. A total of seven studies (2057 patients) satisfied the predefined inclusion criteria, reporting a range of outcomes, including cryosurvival rate (*n* = 3), implantation rate (*n* = 4), positive hCG rate (*n* = 6), clinical pregnancy rate (*n* = 7), ongoing pregnancy rate (*n* = 3), pregnancy failure rate (*n* = 5), live birth rate (*n* = 6), and neonatal outcomes (*n* = 3).

### 3.2 Study and Participant Characteristics

Baseline characteristics of included studies are shown in Table 1. All studies focus on IVF patients, with one study [13] specifically targeting those with recurrent implantation failure (RIF). The patient age across studies is consistent, ranging from early 30s to mid‐30s. The population sizes also vary significantly, with sample sizes ranging from as small as 48 to as large as 355. While most studies report common IVF outcomes like PHR, CPR, and LBR, specific studies provide additional metrics, such as cryosurvival rate or neonatal outcomes.

**TABLE 1:** Baseline characteristics of included studies.

Study	Study design	Country	Year of publication	Patient		Age		Population		Measured outcomes
				Inclusion criteria	Exclusion criteria	FT‐CDB	DFB	FT‐CDB	DFB	
Aytac et al. [8]	Retrospective study	Turkey	2021	FET cycles	PGT, RIF, IVF failure, age ≥ 40	30.9 ± 4.6	30.6 ± 4.5	355	279	Cryosurvival rate, PHR, IR, CPR, LBR, PFR, neonatal outcomes
Xiong et al. [9]	Retrospective study	China	2019	Blastocyst transfer after extended culture	Transfer of one new blastocyst with another cryopreserved blastocyst, slow‐freezing cycles, PGT, frozen–thawed oocytes, cycles that were missing any data	34.15 ± 3.95	34.07 ± 4.14	134	237	PHR, IR, CPR, OGP, LBR, PFR, neonatal outcomes, multiple pregnancy rate
Rahav‐Koren et al. [10]	Retrospective study	Israel	2021	Patients with embryos cryopreserved on Day 3/5	Age > 45, fertility preservation, surrogacy, egg donation, RIF	32.8 ± 5.1	32.3 ± 5.6	224	226	CPR, OGP, LBR, PFR, neonatal outcomes
Tran et al. [11]	Retrospective study	Vietnam	2024	Age 18–45, FET cycles, no uterine interventions or major medical conditions	Uterine factor infertility, uterine surgery, donor cycles, surrogacy, PGT	33.22 ± 4.35	34.31 ± 2.99	58	58	PHR, CPR, OGP, LBR, PFR
Le et al. [12]	Retrospective study	Vietnam	2021	FET cycles	Age > 45, low responders (< 4 oocytes), oocyte donors, endometrial thickness < 7 mm at FET	34.11 ± 4.52	31.71 ± 4.56	52	59	PHR, CPR, IR, no. of survived embryos
Li et al. [13]	Retrospective study	China	2023	Age < 38, RIF, endometrial thickness ≥ 8 mm on transfer day	< 2 cryopreserved embryos, hydrosalpinx, endometriosis, uterine fibroids	32.83 ± 3.48	33.07 ± 3.25	58	166	PHR, IR, CPR, multiple pregnancy rate, PFR, LBR, no. of viable embryos
Önalan et al. [14]	Retrospective study	Turkey	2023	≥ 1 cryopreserved embryo (Day 3/5)	N/A	32.4 ± 4.4	32.2 ± 5.6	103	48	PHR, CPR, LBR

Abbreviations: CPR, clinical pregnancy rate; FET, frozen embryo transfer; IR, implantation rate; LBR, live birth rate; N/A, not available; OGP, ongoing pregnancy rate; PFR, pregnancy failure rate; PGT, preimplantation genetic testing; PHR, positive hCG rate; RIF, recurrent implantation failure.

The Newcastle–Ottawa Scale (Table S2) assessment demonstrated that all studies included in the analysis exhibited high methodological quality. No publication bias was detected by Egger's test (Table S3).

### 3.3 Primary Findings

#### 3.3.1 The FT‐CDB Group Was Associated With Improved Pregnancy Outcomes

**FIGURE 1:**
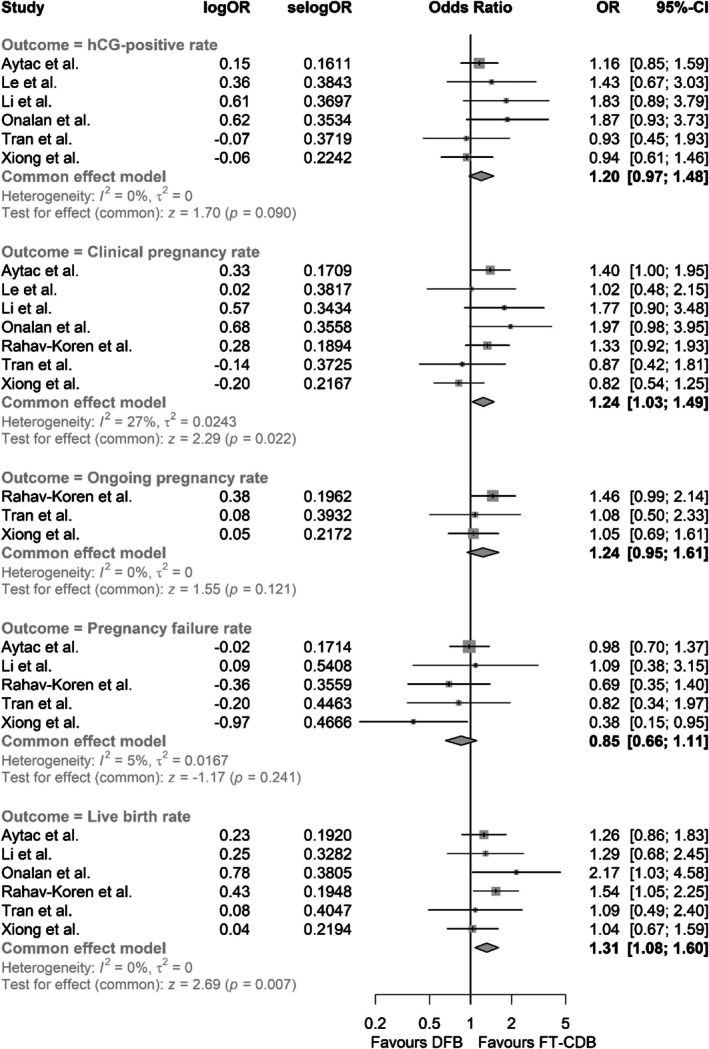
Association between modes of embryo transfer and pregnancy outcomes.

Participants in the FT‐CDB group demonstrated 24% higher odds of clinical pregnancy (OR 1.24, 95% CI 1.03–1.49, *p* = 0.022, *I* ^2^ = 27%) and 31% higher odds of live birth (OR 1.31, 95% CI 1.08–1.60, *p* = 0.007, *I* ^2^ = 0%) compared to those in the DFB group. Furthermore, the FT‐CDB group showed trends toward an improved positive beta‐hCG rate, ongoing pregnancy rate, and a decreased pregnancy failure rate (Figure 1).

#### 3.3.2 The FT‐CDB Transfer Did Not Affect Embryo or Neonatal Outcomes

Additionally, the FT‐CDB group did not affect the cryosurvival rate (OR 0.53, 95% CI 0.06–4.41, *p* = 0.560, *I* ^2^ = 68%) or the implantation rate (OR 1.16, 95% CI 0.75–1.80, *p* = 0.502, *I* ^2^ = 73%, Figure S2).

No significant difference was observed in the birth weights of infants born to mothers in the FT‐CDB group compared to those in the DFB group (MD –87.05 g, 95% CI –293.77 to 119.67, *p* = 0.41, *I* ^2^ = 83%, Figure S3). Given the considerable heterogeneity observed, we conducted additional sensitivity analyses, including influence analysis and outlier removal. Both approaches consistently identified the study by Xiong et al. as the primary contributor to the high heterogeneity. Upon excluding this study, the *I* ^2^ value dropped to 0%, while the result remained statistically non‐significant (MD 11.69 g, 95% CI –64.07 to 87.45, *p* = 0.76, *I* ^2^ = 0%). These sensitivity analyses support the robustness of our findings.

#### 3.3.3 The Superiority of the FT‐CDB Transfer Was Not Affected by Maternal Age, BMI, Duration of Infertility, or Endometrial Thickness

No differences in baseline characteristics regarding maternal age (MD 0.20 years, 95% CI –0.19 to 0.59, *p* = 0.32, *I* ^2^ = 46%), BMI (MD 0.26 kg/m^2^, 95% CI –0.08 to 0.61, *p* = 0.14, *I* ^2^ = 5%), duration of infertility (MD 0.05 years, 95% CI –0.28 to 0.38, *p* = 0.79, *I* ^2^ = 0%), and endometrial thickness (MD −0.05 mm, 95% CI –0.37 to 0.27, *p* = 0.74, *I* ^2^ = 62%) were observed among groups (Table S4).

## 4 Discussion

### 4.1 Summary of Findings

In this study, we synthesized evidence from multiple studies to evaluate whether the FT‐CDB transfer strategy offers advantages in terms of pregnancy outcomes, embryo viability, and birth weights. The FT‐CDB group demonstrated higher rates of clinical pregnancies and live births. Additionally, trends toward improved hCG‐positive rates, ongoing pregnancy rates, and reduced pregnancy failure rates were observed; although these did not reach statistical significance. These results suggest that the FT‐CDB transfer approach may offer advantages in achieving successful pregnancies in IVF treatments.

The analysis also showed that FT‐CDB transfers did not significantly impact embryo or neonatal outcomes. While there was a tendency for a higher cryosurvival rate in the FT‐CDB group, this was not statistically significant; no meaningful differences were observed in infant birth weights between the groups. Furthermore, the benefits of FT‐CDB transfers in pregnancy outcomes were consistent regardless of maternal age, BMI, duration of infertility, or endometrial thickness, indicating that this approach is effective across a broad range of patient characteristics.

### 4.2 Compare With Existing Literature

Our findings align with several previous studies included in this review [8, 10, 14]. Similarly, Rahav‐Koren et al. demonstrated that the FT‐CDB approach significantly increased the likelihood of ongoing pregnancy and delivery rates compared to DFB transfers [10]. In addition, Aytac et al. found higher implantation and clinical pregnancy rates in the FT‐CDB group compared to the DFB group, which supports our findings of improved clinical pregnancy outcomes in the FT‐CDB group [8]. Lastly, findings from Önalanet al. indicated that the FT‐CDB strategy may result in better pregnancy results than the DFB strategy [14]. The strength of these studies lies in their large sample sizes and comparative cohort designs, providing robust evidence that supports our conclusion that the FT‐CDB approach is associated with improved pregnancy outcomes.

Despite these promising results, other studies included in this review found no significant difference in pregnancy between the FT‐CDB and DFB groups [9, 12, 13]. For instance, the study by Xiong et al. demonstrated comparable pregnancy outcomes between the two groups [9]. Le et al. also found that extending the culture of cleavage embryos to the blastocyst stage did not outperform direct frozen blastocyst transfer [12]. Finally, the study of Li et al. indicated no significant difference in clinical pregnancy rate between the FT‐CDB and DFB groups among RIF patients [13]. These findings suggest that, while culturing cleavage‐stage embryos to the blastocyst stage may offer advantages in certain cases, this strategy does not consistently outperform direct frozen blastocyst transfer.

### 4.3 Interpretation

The improved outcomes observed in FT‐CDB may reflect a combination of biological and technical factors. Extended post‐thaw culture allows for the natural selection of embryos capable of continued development, enriching for a more robust population with higher implantation potential. However, it is important to acknowledge that the DFB group also undergoes a selection process, as only embryos that reach the blastocyst stage and survive vitrification are included for transfer. Thus, both FT‐CDB and DFB strategies impose selective pressures at different developmental checkpoints; attributing enhanced outcomes solely to post‐thaw culture may oversimplify the underlying biology.

Vladimirov et al. proposed a hypothesis suggesting that freeze–thaw exposure may induce adaptive responses that enhance embryonic developmental competence, a concept known as hormesis [15]. However, their study also reported a 14% higher miscarriage rate following thawed blastocyst transfers compared to thawed cleavage‐stage transfers. The authors suggest this may be due to the detoxified effect and increased trophectoderm activity post‐thaw, which could facilitate the implantation of aneuploid blastocysts. In contrast, cleavage‐stage embryos with such abnormalities are more likely to arrest before reaching the blastocyst stage. Therefore, while hormetic stress responses may play a role in the outcomes observed in the FT‐CDB group, additional biological mechanisms are likely involved; further studies are needed to fully understand these complex processes.

On the other hand, technical challenges such as the presence of the blastocoel cavity in blastocysts affect cryopreservation survival, and interventions like artificial shrinkage can improve outcomes; although such procedures are not widely practiced [16]. These biological and technical factors together may contribute to the differential reproductive outcomes observed between cleavage‐stage and blastocyst‐stage cryopreservation.

An additional consideration with the FT‐CDB approach is the potential for a high cancellation rate [11, 13]. Unlike the DFB group, where embryos are cultured to the blastocyst stage prior to freezing, the FT‐CDB approach involves thawing and culturing a limited number of cleavage‐stage embryos, typically selected based solely on morphology at the cleavage stage. However, the predictive value of a one‐time morphology assessment at this stage for blastocyst development is low, resulting in greater variability in embryo progression [17]. This increases the risk of cycle cancellations, where embryos fail to reach the blastocyst stage and are unsuitable for transfer. Such cancellations can delay pregnancy and impose emotional and financial burdens on patients.

From an operational perspective, the FT‐CDB strategy also increases the workload for embryologists, as more time is spent monitoring and culturing embryos that may not ultimately result in a viable transfer [18]. This not only adds to the time and resource demands within IVF centers but also underscores the potential inefficiency of handling thawed embryos that are unlikely to progress to the blastocyst stage. Therefore, optimizing the thawing process, refining embryo selection, and improving culture conditions are essential steps in reducing the risk of cycle cancellations [19, 20, 21].

In some cases, the FT‐CDB transfer strategy may not be suitable for cleavage‐stage embryos with high fragmentation or low‐quality potential, as these embryos tend to have a lower survival rate after thawing and are less likely to progress to the blastocyst stage [22]. In such instances, the DFB transfer approach becomes a more feasible option, benefiting from a natural selection mechanism [22]. In addition, when all thawed cleavage embryos successfully reach the blastocyst stage, the recommendation to transfer only one good‐quality embryo means that surplus blastocysts must undergo cryopreservation a second time. This additional round of freezing and thawing can reduce the developmental potential of the surplus embryos [23]. After reviewing the included studies, two articles reported data on the number of thawed cleavage‐stage embryos, the number of usable blastocysts, and the number of blastocysts transferred [12, 13]. From this data, it is clear that there are surplus embryos, as the number of usable blastocysts exceeds the number of blastocysts transferred. This indicates embryo wastage and the potential for re‐cryopreservation, which could further impact the embryos' development. Furthermore, this process presents a significant challenge for embryologists, who must manage both the thawing and culturing procedures, which can lead to burnout, as noted in recent studies, particularly in the UK and U.S. settings [24]. In contrast, the DFB approach reduces the overall workload for embryologists by minimizing the need to handle embryos that may not survive.

### 4.4 Strengths and Limitations

To the best of our knowledge, this is the first systematic review and meta‐analysis comparing FT‐CDB and DFB transfer strategies, making it a valuable contribution to the existing literature. However, several limitations must be acknowledged.

Firstly, only seven retrospective studies were included, all of which relied on per‐protocol (PP) analyses; no randomized controlled trials (RCTs) were available. While PP remains the conventional approach in IVF research, it may introduce selection bias—particularly in studies where outcomes depend on which embryos survive and are selected for transfer. Most of the included studies reported outcomes per embryo transfer, excluding canceled cycles, which could lead to an overestimation of treatment efficacy and limit generalizability to real‐world clinical practice. In contrast, intention‐to‐treat (ITT) analysis, though rarely applied in this field, offers a more robust estimate of effectiveness. Although ITT‐based RCTs would help minimize selection bias, they present practical and ethical challenges. For example, randomizing patients to different embryo transfer strategies without considering individual prognoses or embryo development potential could raise concerns about fairness and patient safety. Additionally, withholding preferred transfer protocols may not be acceptable in some clinical settings. To address these issues, future trial designs could employ stratified randomization based on patients' baseline characteristics, IVF history, and embryo development patterns, as assessed through time‐lapse imaging. This would help create more comparable patient groups, improving balance across study arms and reducing bias. By maintaining ethical standards while mitigating bias, these strategies would strengthen the reliability of ITT‐based RCTs. Future research should prioritize these challenges to provide more robust evidence.

Secondly, the inconsistent reporting of IVF indications across the included studies presents a limitation. Clinical characteristics such as tubal factor, male factor, unexplained infertility, endometriosis, and RIF can influence the outcomes. The lack of detailed IVF indication information makes it difficult to determine whether observed differences in outcomes are due to the embryo transfer strategy itself or differences in baseline fertility status. Additionally, the included studies varied in sample sizes, patient populations, and were published over a long period. This variability could introduce inconsistencies in clinical practices, patient demographics, and technological advancements, which may affect the results.

Lastly, the improved outcomes observed in the FT‐CDB group may be influenced by both the extended culture strategy and selection bias. Since embryos selected for extended culture are morphologically superior at the cleavage stage, this may skew the outcomes in favor of the FT‐CDB group. Another potential limitation is the variation in vitrification protocols across the included studies, which could result in differences in cryosurvival rates. Such variations may significantly influence pregnancy outcomes, as embryo survival is critical for successful embryo transfer [25]. These sources of bias are difficult to control in retrospective studies, and they represent an inherent limitation of the current evidence base.

### 4.5 Wider Implication

While the FT‐CDB transfer strategy shows promising potential for improving pregnancy outcomes in this study, its application must be carefully evaluated on a case‐by‐case basis, considering the specific conditions of each IVF center. The variability in embryo quality, the challenges associated with multiple rounds of cryopreservation and thawing, the increased workload for embryologists, and the risk of cycle cancellation emphasize the need for more refined protocols and patient‐specific selection criteria. Future research should focus on well‐designed, multicenter RCTs with ITT analysis to provide a clearer comparison between the FT‐CDB and DFB strategies.

## 5 Conclusions

In conclusion, transferring blastocysts derived from frozen–thawed cleavage embryos is associated with higher odds of clinical pregnancy and live birth compared to frozen–thawed blastocyst transfers. However, this study also highlights the risks of cycle cancellation and the increased workload for embryologists associated with the transfer of blastocysts derived from frozen–thawed cleavage embryos. Therefore, the application of this approach should be carefully considered.

## Conflicts of Interest

The authors declare no conflicts of interest.

## Supporting information


**Data S1:** rmb212673‐sup‐0001‐DataS1.docx.

## Data Availability

The data extracted from the original studies are publicly accessible. The datasets generated or analyzed during this study are available from the corresponding author upon reasonable request.
