# Supplementary material for: Reproductive Outcomes of Transferring Blastocysts Derived From Frozen–Thawed Cleavage Embryos: A Systematic Review and Meta‐Analysis
Source: Reprod Med Biol. 2025 Aug 7;24(1):e12673. doi: 10.1002/rmb2.12673 (PMC12329422; doi:10.1002/rmb2.12673)
Supplement: Supplementary file 1 — Data S1: rmb212673‐sup‐0001‐DataS1.docx. [file RMB2-24-e12673-s001.docx]

**Reproductive Outcomes of Transferring Blastocysts Derived from Frozen-Thawed Cleavage Embryos: A Systematic Review and Meta-analysis**

**Supplementary Materials**

**Table S1.** Search strategy (all fields included) 2

**Table S2.** Study quality assessment using the Newcastle-Ottawa tool 3

**Table S3.** Publication bias analysis performed using Egger’s test 4

**Table S4.** Differences in baseline characteristics between the FT-CDB and DFB groups 5

**Figure S1.** Preferred Reporting Items for Systematic Reviews and Meta-Analyses flowchart 6

**Figure S2.** Association between modes of embryo transfer and embryo outcomes 7

**Figure S3.** Forest plot comparing mean birth weight between the FT-CDB and DFB groups 8

**Table S1.** Search strategy (all fields included)

| **PubMed** | | |
| --- | --- | --- |
| **No** | **Query** | **Results** |
| #1 | Blastocyst OR “day 5 embryo” | 45040 |
| #2 | Cleavage-stage OR “day 3 embryo” | 4398 |
| #3 | “Fertilization rate” OR “blastulation rate” OR “cryosurvival rate” OR “pregnancy outcome” OR “implantation rate” OR “clinical pregnancy rate” OR “ongoing pregnancy rate” OR “live birth rate” OR “pregnancy failure rate” | 78945 |
| #4 | #1 AND #2 AND #3 | 478 |
| **EMBASE** | | |
| #1 | Blastocyst OR “day 5 embryo” | 43556 |
| #2 | Cleavage-stage OR “day 3 embryo” | 4232 |
| #3 | “Fertilization rate” OR “blastulation rate” OR “cryosurvival rate” OR “pregnancy outcome” OR “implantation rate” OR “clinical pregnancy rate” OR “ongoing pregnancy rate” OR “live birth rate” OR “pregnancy failure rate” | 118609 |
| #4 | #1 AND #2 AND #3 | 994 |
| **Scopus** | | |
| #1 | Blastocyst OR “day 5 embryo” | 104835 |
| #2 | Cleavage-stage OR “day 3 embryo” | 17089 |
| #3 | “Fertilization rate” OR “blastulation rate” OR “cryosurvival rate” OR “pregnancy outcome” OR “implantation rate” OR “clinical pregnancy rate” OR “ongoing pregnancy rate” OR “live birth rate” OR “pregnancy failure rate” | 296735 |
| #4 | #1 AND #2 AND #3 | 6553 |
| **Web of Science** | | |
| #1 | Blastocyst OR “day 5 embryo” | 33334 |
| #2 | Cleavage-stage OR “day 3 embryo” | 3622 |
| #3 | “Fertilization rate” OR “blastulation rate” OR “cryosurvival rate” OR “pregnancy outcome” OR “implantation rate” OR “clinical pregnancy rate” OR “ongoing pregnancy rate” OR “live birth rate” OR “pregnancy failure rate” | 29839 |
| #4 | #1 AND #2 AND #3 | 460 |

**Table S2.** Study quality assessment using the Newcastle-Ottawa tool

|  | Selection (max 4 stars) | Comparability (max 2 stars) | Outcome (max 3 stars) |
| --- | --- | --- | --- |
| Aytac et al. | 4/4 | 2/2 | 2/2 |
| Xiong et al. | 4/4 | 2/2 | 2/2 |
| Rahav‑Koren et al. | 4/4 | 2/2 | 2/2 |
| Tran et al. | 4/4 | 2/2 | 2/2 |
| Le et al. | 4/4 | 2/2 | 2/2 |
| Li et al. | 4/4 | 2/2 | 2/2 |
| Onalan et al. | 4/4 | 2/2 | 2/2 |

**Table S3.** Publication bias analysis performed using Egger’s test

| **Outcome** | **Intercept** | **SE** | **Slope** | ***t* test** | **df** | **p value** |
| --- | --- | --- | --- | --- | --- | --- |
| Cryosurvival rate | −2.10 | 1.76 | 1.29 | −1.19 | 1 | 0.44 |
| Implantation rate | 1.32 | 5.10 | −0.15 | 0.26 | 2 | 0.82 |
| hCG-positive rate | 1.24 | 1.11 | −0.12 | 1.11 | 4 | 0.33 |
| Clinical pregnancy rate | 0.04 | 1.53 | 0.21 | 0.03 | 5 | 0.98 |
| Ongoing pregnancy rate | −1.19 | 2.31 | 0.48 | −0.51 | 1 | 0.70 |
| Pregnancy failure rate | −1.18 | 0.99 | 0.16 | −1.19 | 3 | 0.32 |
| Live birth rate | 0.51 | 1.36 | 0.16 | 0.37 | 4 | 0.73 |

**Table S4.** Differences in baseline characteristics between the FT-CDB and DFB groups

| **Characteristics** | **Number of studies** | **Sample size** | **Mean difference** | **95% CI** | **I^2^** | **p value** |
| --- | --- | --- | --- | --- | --- | --- |
| Maternal age | 7 | 2057 | 0.20 years | -0.19 to 0.59 | 46% | 0.32 |
| Maternal body mass index | 6 | 1946 | 0.26 kg/m^2^ | -0.08 to 0.61 | 5% | 0.14 |
| Duration of infertility | 4 | 1340 | 0.05 years | -0.28 to 0.38 | 0% | 0.79 |
| Endometrial thickness | 5 | 1491 | -0.05 mm | -0.37 to 0.27 | 62% | 0.74 |


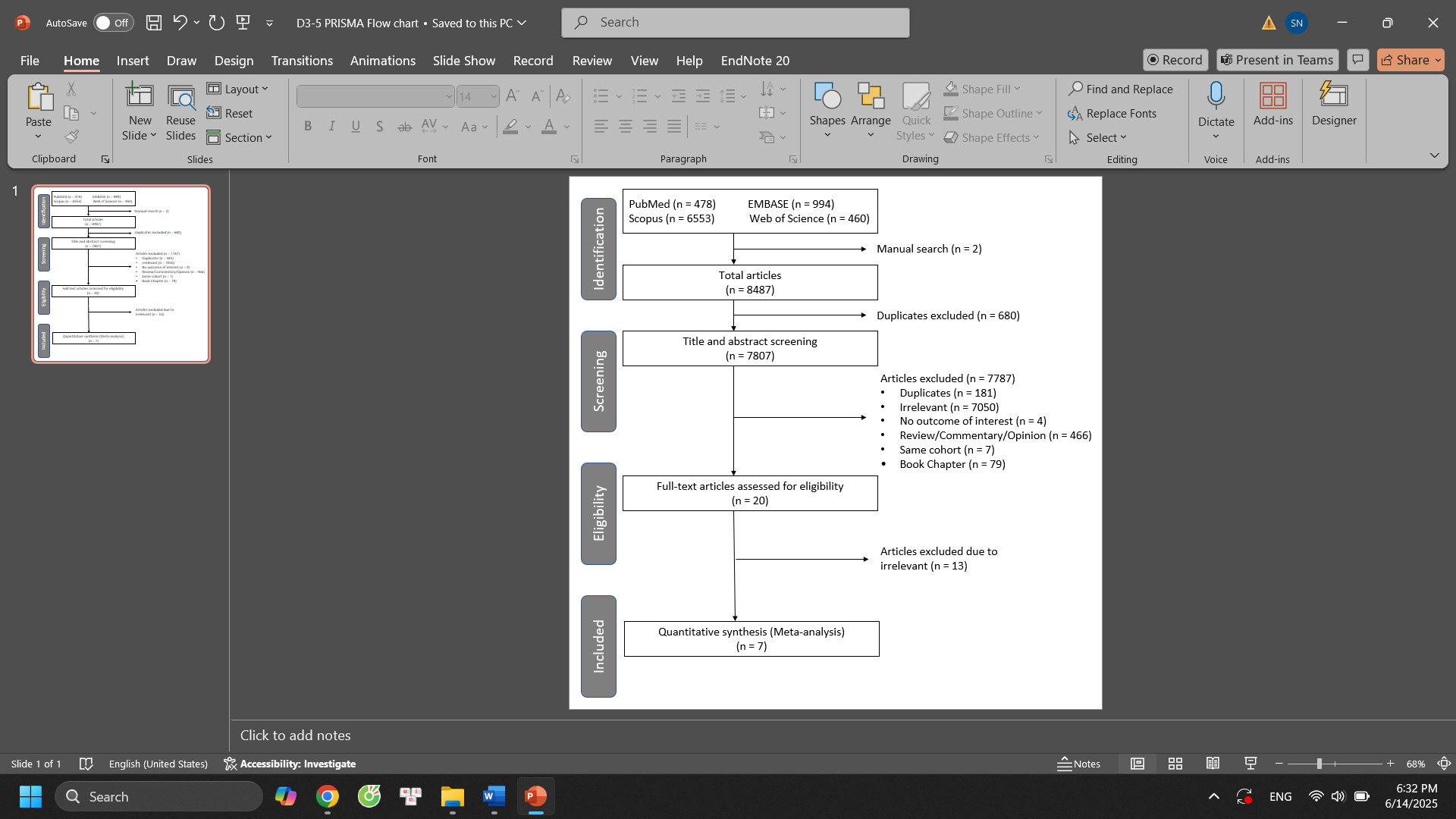
**Figure S1.** Preferred Reporting Items for Systematic Reviews and Meta-Analyses flowchart


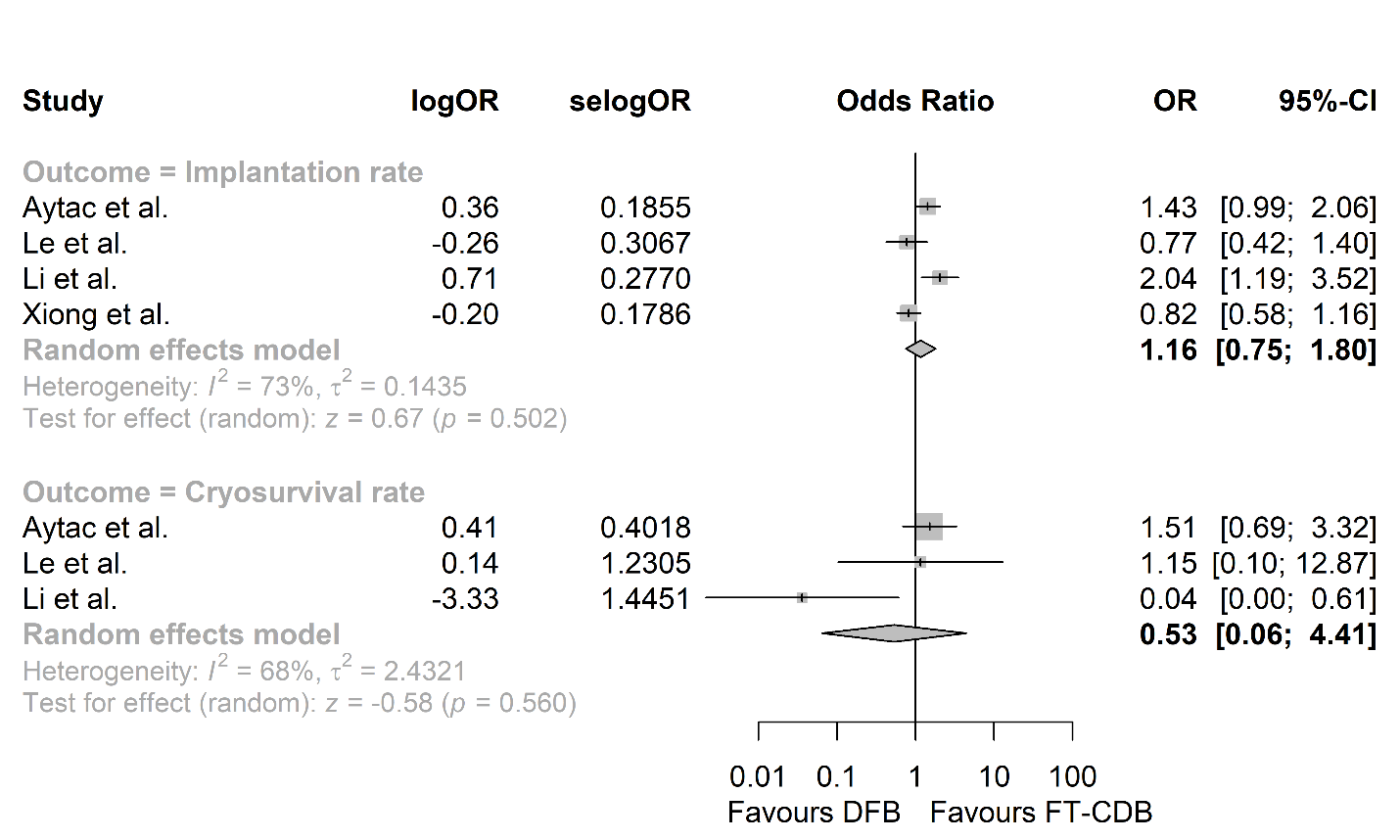


**Figure S2.** Association between modes of embryo transfer and embryo outcomes


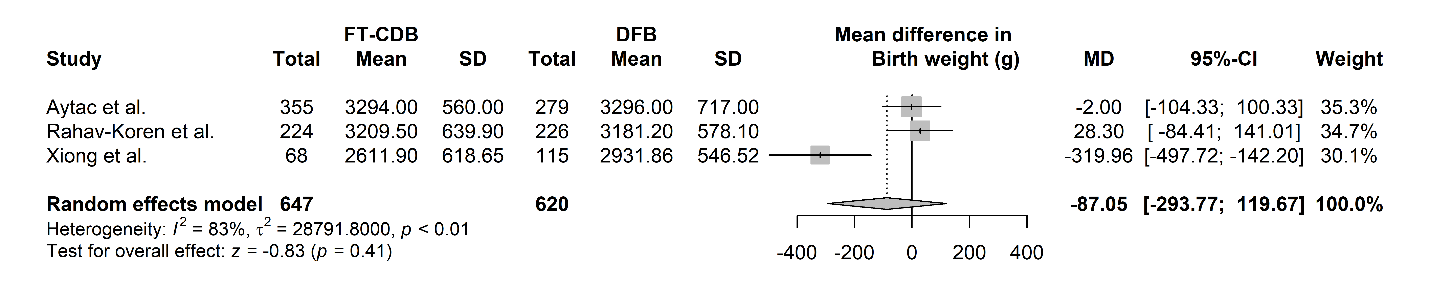


**Figure S3.** Forest plot comparing mean birth weight between the FT-CDB and DFB groups.

Positive values indicate higher birth weight in the FT-CDB group; negative values indicate higher birth weight in the DFB group
